# Supplementary material for: Serological Screening for Antibodies against SARS-CoV-2 in Dutch Shelter Cats
Source: Viruses. 2021 Aug 18;13(8):1634. doi: 10.3390/v13081634 (PMC8402678; doi:10.3390/v13081634)
Supplement: Supplementary file 1 [file viruses-13-01634-s001.zip › viruses-1293679-supplementary.pdf]

Appendix A1 – Questionnaire for the participating animal shelters in the seroprevalence study of antibodies against SARS-CoV-2 in Dutch shelter cats.

General information

Name animal shelter:
Address animal shelter:
Date of completion of this form:
Name and position in shelter organisation of respondent:
Name of shelter veterinarian:

Details animal shelter

Capacity for annual animal care:
Which animal species are taken in:

Human and animal contact with SARS-CoV-2

Does shelter receive pet(s) from COVID-19 environment?
Number of pets taken in from COVID-19 environment:
Number of cats taken in from COVID-19 environment:

Housing and care of cats from COVID-19 environments

In which shelter department are cats from COVID-19 environments housed?
Does care for cats from COVID-19 environment differ from the general feline care?
Is care different in terms of housing?
Is care different in numbers of staff members?
Is care different in use of protective measures taken by staff members or other personnel?
What kind of protective measures are taken?
